# Interactive Medical Image Segmentation Using Snake and Multiscale Curve Editing

**DOI:** 10.1155/2013/325903

**Published:** 2013-12-12

**Authors:** Wu Zhou, Yaoqin Xie

**Affiliations:** Shenzhen Key Laboratory for Low-Cost Healthcare, Shenzhen Institutes of Advanced Technology, Chinese Academy of Sciences, Shenzhen 518055, China

## Abstract

Image segmentation is typically applied to locate objects and boundaries, and it is an essential process that supports medical diagnosis, surgical planning, and treatments in medical applications. Generally, this process is done by clinicians manually, which may be accurate but tedious and very time consuming. To facilitate the process, numerous interactive segmentation methods have been proposed that allow the user to intervene in the process of segmentation by incorporating prior knowledge, validating results and correcting errors. The accurate segmentation results can potentially be obtained by such user-interactive process. In this work, we propose a novel framework of interactive medical image segmentation for clinical applications, which combines digital curves and the active contour model to obtain promising results. It allows clinicians to quickly revise or improve contours by simple mouse actions. Meanwhile, the snake model becomes feasible and practical in clinical applications. Experimental results demonstrate the effectiveness of the proposed method for medical images in clinical applications.

## 1. Introduction

Medical image segmentation is of great importance in providing noninvasive information for human body structures that helps clinicians to visualize and study the anatomic structures, track the progress of diseases, and evaluate the need for radiotherapy or surgeries [1]. Even though the research and application of medical images techniques are expanding rapidly, accurate segmentation of medical images meets many challenges in clinical applications due to the inhomogeneity of anatomical structures, low contrast, noise and occlusions. All these challenges make the medical image segmentation difficult in clinical applications. To overcome these challenges, many segmentation methods have been developed and reported in the literature [2]. However, no segmentation method works well for all the applications, and various approaches have been explored for each computer-aided diagnosis (CAD) problem. Furthermore, a particular segmentation may work well for one, but not for another subject, or only on certain images of one anatomical structure. Therefore, segmentation or delineation is still a very active research field and how to design an optimal segmentation approach that fulfills the necessities of clinical applications is extremely essential for medical clinicians.

Generally, medical image segmentation is manually done by experts or clinicians slice by slice to obtain accurate boundary information of the regions of interest (ROIs) [3]. Manual techniques allow users to outline structures using software such as the ITK-SNAP [4]. Manual segmentation may be accurate, but time consuming and tedious for users. More seriously, it is cause of interobserver variation or bias. A number of computer-aided segmentation techniques have been proposed for medical images, which can usually be distinguished as semiautomatic or fully automatic methods. Semiautomatic techniques may allow the user to have some control or input into the segmentation process, combined with some automatic process using computer algorithms. Semiautomatic approaches based on thresholding [5], region growing [6], and deformable models [7–9] are well considered in numerous applications. Fully automatic techniques require no user input and often make use of some prior knowledge from the anatomy being segmented to produce the segmentation or delineation, and two examples of these approaches are atlas-based segmentation [10] and statistical shape models [11].

Although a lot of automatic or semiautomatic image segmentation approaches have been proposed, few of them can fulfill the necessities of applications in terms of accuracy and efficiency. Both thresholding and region growing methods are relatively straightforward for automatic segmentation, and they work only using the intensities in images and do not impose constraint on the shape of resulting delineation objects [3]. One contour detection method used edge following algorithm based on intensity gradient and texture gradient features proposed for medical images [12], but it was not promising for blur edges or low contrast medical images. In addition, deformable models, such as active contour models or snakes [7], can move and deform the initial delineation according to an energy term. Because of their ability to approximate complex shapes, snakes have been used in many image analysis applications, including nonrigid motion analysis [8] and object tracking [13]. Snakes have been used in segmentation of volumetric images. After segmenting image slices individually, regions obtained in the slices are stacked to form a volumetric region [3, 14]. However, the optimization of parameters in minimizing the energy is generally slow for applications, due to iterative adjustment of the contour for energy minimization. They require careful initialization and it may be difficult for them to achieve initialization invariance and robust convergence [2]. This is really the problem when segmenting objects with complex geometries and shapes in medical images.

Generally, snakes work well where there are clear defined edges in the image and the shape of the object is reasonably smooth, since sharp edges will be smoothed out by the snake's internal energy, which resists high curvature [15, 16]. However, the desired object boundary may be unclear or even partly missing in many medial images. Therefore, the final results of snakes for clinical medical images are subject to contain boundaries errors. It is evidently clear that initialization invariance is particularly difficult to achieve for active contour methods. More recent attempts, such as [17, 18], showed promising but limited success. Recently, Xie [19] presented an initialization-invariant edge based active contour model, which provides great freedom in contour initialization. However, it is also demonstrated that very time-consuming and overwhelming noise interference will inevitably degrade the performance. Overall, active contour models still have difficulties in handling the boundary complexities, weak edges, and image noise, which are very common in clinical medical images [1]. All these factors make active contour models impractical currently in clinical applications. Basically, the clinicians are concerned with the robust and reliability of segmentation methods in applications. Once segmentation contains errors in clinical applications, it should also provide a means to improve the segmentation results.

To this end, a novel and simple approach of interactive contour delineation is proposed, which is combining the snake model and multiscale curve editing to obtain promising results for clinical applications. Initially, a region boundary which covers the interesting object is drawn manually by the user in the image. To reduce the evolution time of snake model and make it work properly, the initial boundary can be freely revised to be close to the actual boundary of ROIs. Then, snake model is used automatically for contour delineation to make the manual process accurate. Once the evolution of the snake mode is stopped, the final contour can be revised optionally in a hierarchical multiscale manner to reduce delineation errors. In the multiscale revision process, firstly uniformly large spacing of control point mesh is generated when delineation errors of final contour of the snake model are large. After manual revision with related control points, the overall delineation errors will be reduced. In order to refine the results further, the control point mesh is progressively refined by clinicians. In this case, the control point mesh at level *i* is refined by inserting new control points to create the control point mesh at level *i* + 1. Therefore, the control point spacing is halved at every step. With the revision of control point at different levels, the final deformation field will be generated to make the revised contour coincide with the actual contour. The multiscale revision process will correct errors of the snake model and can revise very complicated contours.

This paper is organized as follows.  Section 2 describes the material and the proposed contour delineation technique. In Section 3, we show the experimental results on kinds of medical images, and some discussions are given.  Section 4 concludes this paper.

## 2. Methods

### 2.1. The Framework of the Hybrid Method

**Figure 1:**
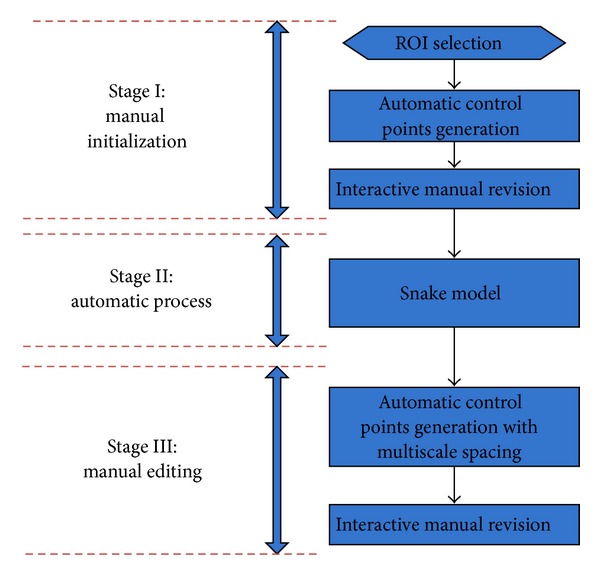
The framework of proposed contour delineation algorithm.

The purpose of the research is to make image segmentation or contour delineation simple and fast in clinical applications. We explore snake model and curve editing to devise a contour delineation algorithm that consists of manual process and automatic process. As shown in Figure 1, the proposed framework of contour delineation algorithm consists of three steps. First is the manual process that the user selects ROIs and manually revises automatically generated control points with mouse action. Second is the automatic process that the contour refinement is achieved by the snake model. Last is the process of manual editing to revise the contour by dragging control points with multiscale spacing. Our proposed technique preserves the advantages of fully automatic techniques and clinical manual techniques. Meanwhile, skilled clinicians or doctors can also incorporate their valuable experiences in the process of manual revision to generate promising results. The stage of automatic snake model is devised to make the manually generated contour more accurate, and it can also eliminate interobserver variation or bias. Occasionally, the automatic process with snake model may contain errors due to the complicated anatomic structures or overwhelming noise in images. So the manual editing in the last stage is often necessary and required in clinical applications. The contribution of our work is developing a complete framework for medical image segmentation in clinical application. The free form drawing and revision make the segmentation process accurate and robust. The adjustment of initialization and correcting errors for the snake model make it practical and robust for clinical applications. We will illustrate the proposed technique in the next sections in detail.

### 2.2. Hermite Cubic Curve for Manual Revision

Curve fitting methods are described that can accurately represent the region boundary with a parametric curve from above generated control points. Thus, the parametric curve generated by control points will replace the initial region boundary, generated by mouse click for further processing. Since the process of manual revision should be very fast and convenient for users, the selection of curve fitting methods is important. The main attributes are that they should be easy to compute and are stable. Actually, a number of interpolation or approximation methods have been proposed in the literature. We choose Hermite cubic curve [20] for manual refinement due to its simplicity and smoothness.

Hermite cubic curve is a powerful tool to smoothly interpolate between key points. Given *P*0 and *P*1 represent the starting and ending points of the curve, and *u*0 and *u*1 represent tangent to how the curve leaves the start point and endpoint, respectively. Four Hermite basis functions are as follows:

(1) $$\begin{array}{c} h1\left(s\right)=2s^{3}-3s^{2}+1,\\ h2\left(s\right)=-2s^{3}+3s^{2},\\ h3\left(s\right)=s^{3}-2s^{2}+s,\\ h4\left(s\right)=s^{3}-s^{2}. \end{array}$$

These 4 vectors *P*0, *P*1, *u*0, and *u*1 are simply multiplied with above 4 Hermite basis functions and added together. Then, the general form of Hermite curve is

(2) $$\begin{array}{c} P\left(s\right)=h1\left(s\right)P0+h2\left(s\right)P1+h3\left(s\right)u0+h4\left(s\right)u1, \end{array}$$

where scale *s* is to go from 0 to 1 with spacing Δ*s*. In general, Δ*s* is 0.1 in our experiments. 10 points will be generated for each segment between two control points, and the connection of these 10 points will be the new digital curve that encloses the initial contour or boundary.

**Figure 2:**
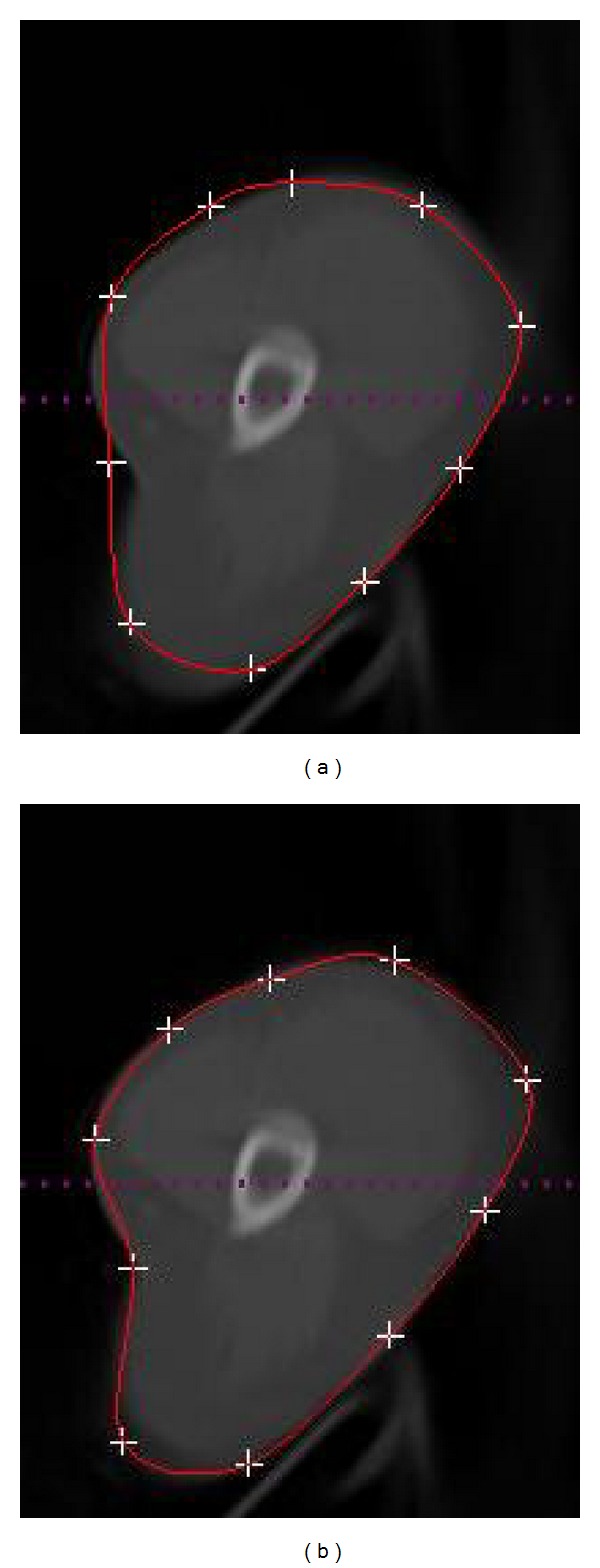
Initial drawing and interactive calibration results for the clinical CT image.

In our experiments of manual revision, all control points can be clicked on and dragged to alter the curves appearance. When one control point is selected for dragging, other control points will not react to dragging. Once the new revised position of the selected control point is determined by left mouse dragging, the new curve will be fitted again by Hermite cubic curve and displayed to replace the former boundary. The process of fitting Hermite cubic curve is real-time with the movement of selected control points by mouse dragging. It is worthwhile to note that the process of manual revision is locally controlled, which means that the movement of control points only affects the local area. This makes it more convenient and efficient than methods such as the linear interpolation or cubic interpolation, where the motion of a single control point affects the whole shape of the curve.  Figure 2 shows the results of Hermite cubic curve for manual revision. The revision of several control points will make the curve enclose the actual boundary, and this process is fast and convenient for clinicians.

### 2.3. Snake Model for Automatic Contour Delineation

Active contour or snakes [8] are used heavily for boundary delineation or edge detection in medical images. A snake is defined as an energy minimization spline whose energy depends on its shape and location within the image. Shape of the snake is controlled by the internal forces and external forces. The external force guides the snake towards the features in the image, and internal force acts as smoothing constraint for the snake. Let the vector *p*(*s*) = (*x*(*s*), *y*(*s*)) is the parametric representation of the snake where the value of *s* goes from 0 to 1. The energy function that we want to minimize is defined and represented as follows [8]:

(3) $$\begin{array}{c} E_{\text{Snake}}=\overset{1}{\underset{0}{\int }}E_{\mathrm{int}}\left(p\left(s\right)\right)+E_{\text{ext}}\left(p\left(s\right)\right)ds. \end{array}$$

*E*_int⁡_ is the internal forces that forces the snake to be small and smooth. *E*_ext_ is an external energy for the snake finding the edges of an object in the image. A common external energy is the inverse of the gradient magnitude, in other words, the low energies at the location of the edges, the higher energies everywhere else. Here the objective is to find such *p*(*s*) so that the total energy of the snake is minimized. The internal energy is defined as

(4) $$\begin{array}{c} E_{\mathrm{int}}=\frac{1}{2}\left\{\alpha {\left|p^{\prime }(s)\right|}^{2}+\beta {\left|p^{\prime \prime }(s)\right|}^{2}\right\}, \end{array}$$

where |*p*′(*s*)| is the magnitude of the first derivative, which is larger for longer snakes. |*p*′′(*s*)| is the magnitude of the second derivative, which is larger for sharper bends. The first part keeps the snake short and the second part keeps it straight. The two parameters *α* and *β* define the relative importance of these two terms, which are usually constant. Given a gray-level image *I*(*x*, *y*), typical external energy *E*_ext_ designed to lead an active contour toward step edges that are defined as follows:

(5) $$\begin{array}{c} E_{\text{ext}}=-{\left|\nabla \left(G_{\sigma }\left(x,y\right)\right)*I\left(x,y\right)\right|}^{2}, \end{array}$$

where *G*_*σ*_(*x*, *y*) is a two-dimensional Gaussian function with standard deviation *σ* and ∇  is the gradient operator. It is easy to find that larger *σ* will cause the boundaries to become blurry and distorted. However, such large *σ* is often necessary in order to make the external energy large enough to pull the snake towards these edges.

To minimize the energy function we use the Euler-Lagrange equation

(6) $$\begin{array}{c} \alpha p^{\prime \prime }\left(s\right)-\beta p^{\prime \prime \prime \prime }\left(s\right)-E_{\text{ext}}\left(p\left(s\right)\right)=0. \end{array}$$

Above equation is solved by using the gradient decent method. One converts the snake *p* into a function of time *t*, and replaces the 0 with the partial derivative of *p* to time

(7) $$\begin{array}{c} \frac{\partial p\left(s,t\right)}{\partial t}=\alpha p^{\prime \prime }\left(s,t\right)-\beta p^{\prime \prime \prime \prime }\left(s,t\right)-E_{\text{ext}}\left(p\left(s,t\right)\right). \end{array}$$

When the snake has converged to a minimum and the solution *p*(*s*, *t*) stabilizes, its derivative to time will be zero and we achieve a solution of above Euler-Lagrange equation.

**Figure 3:**
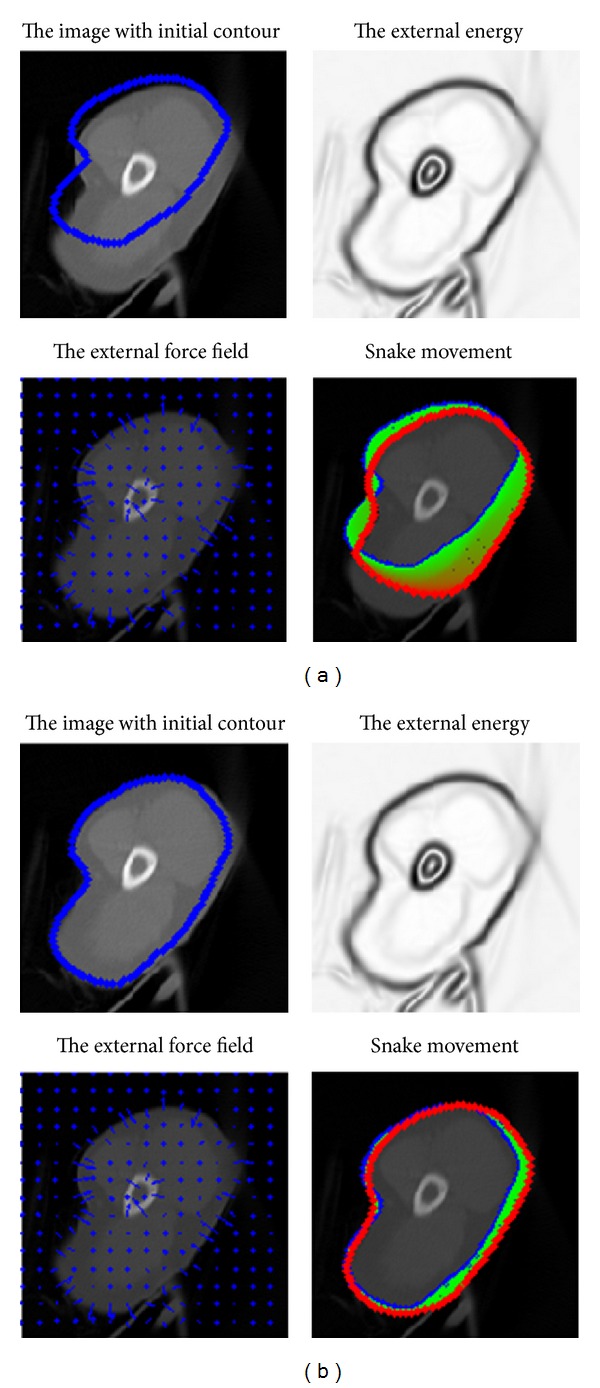
The results of the snake model with different initial contour. (a) The initial contour is far away from the actual contour. (b) The initial contour is close to the actual contour. Note that the initial contour in (b) is generated by dragging control points of the initial contour in (a).

**Figure 4:**
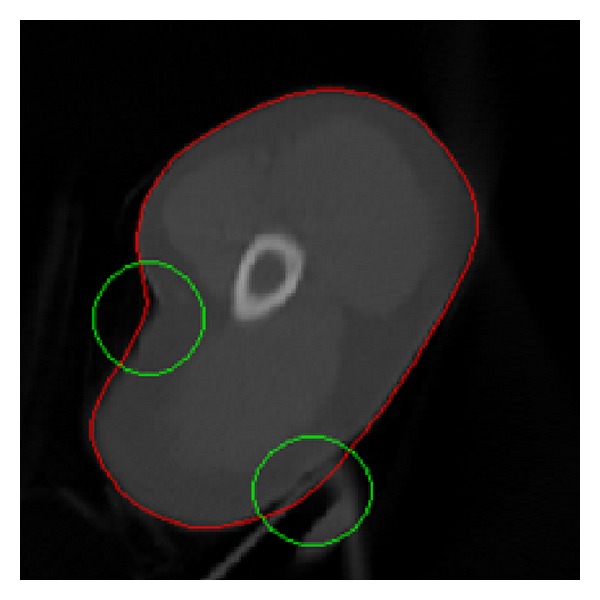
The result of the snake model. Red shows the obtained contour by the snake model. Boundaries in green circles contain errors due to the sharp edges with high curvature and blurred boundary, respectively.

In general, one needs to initialize the snake close to the final solution. If the snake is initialized “too far” from the object boundary, it is possible that the contour may not be able to converge onto object boundary. We experimentally find that boundaries errors are very common from the results of the snake model for clinical applications.  Figure 3 shows the experimental results of the snake model with different initial contours. If the initial contour is far away from the actual contour, the results would be unsatisfying. Therefore, the process of manual initialization is necessary to revise the initial contour to be close to the actual contour. Meanwhile, the time consumed will be greatly reduced if the initial contour is close to the actual contour. In addition, sharp edges will be smoothed out by the snake's internal energy, which resists high curvature. Therefore, the final results of snakes for clinical medical images are subject to contain boundaries errors.  Figure 4 shows the experimental result of the snake model with good initial contour and optimal parameters. However, boundaries in green circles contain errors due to the sharp edges with high curvature and the blurred boundary.

### 2.4. Manual Editing with Multiscale Control Points

Generally, most of boundaries obtained from the snake model can be correct and few segments may contain errors. If the results of the snake model are perfect, there is no need for further multiscale curve editing. The manual editing should only revise boundary errors in limited areas and other boundaries areas should be kept. Therefore, the manual editing should be local control. In other words, operating one boundary areas should not affect other boundary area. Furthermore, the shape of the boundary may be unknown and very complicated that fixed control points may not be flexible for revision. To this end, we design a hierarchical multiscale approach to generate control points for revision with Hermite cubic curves.

**Figure 5:**
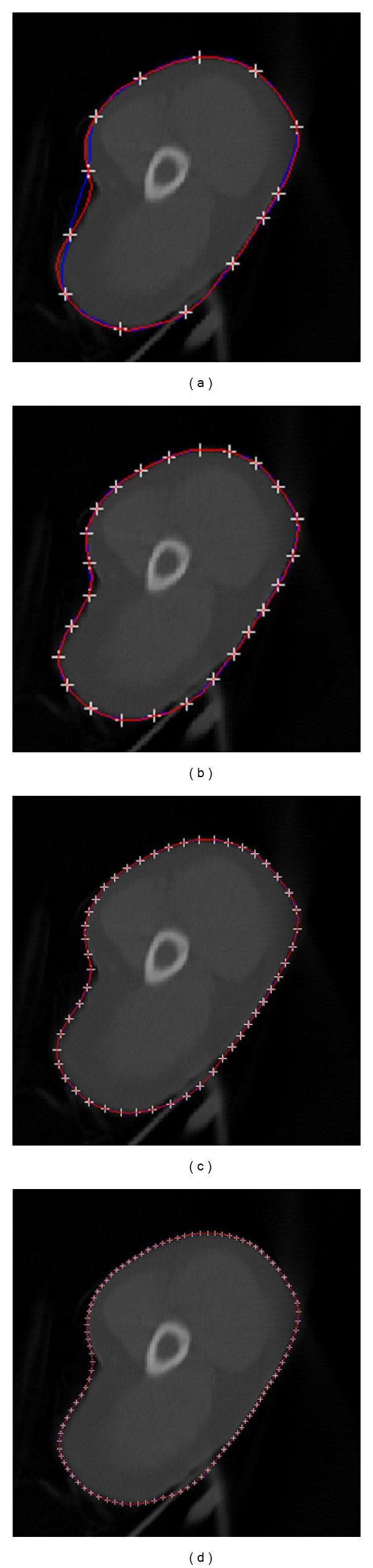
A hierarchical multiscale approach to generate uniformly spaced control points for manual editing: (a) first level with 13 control points, (b) second level with 25 control points, (c) third level with 50 control points, and (d) fourth level with 100 control points. White cross shows the uniformly spaced control points. Red color shows the contour obtained from the snake model, and blue color shows the curve generated by Hermite cubic curve with associated control points.

Figure 5 shows the control point generation by the proposed hierarchical multiscale approach. On the first level, only 13 control points are generated from the contour obtained by the snake model. Due to the complex shape of the boundary, 13 points on the first level are not enough to describe the contour well. In Figure 5(a) the fitted Hermite cubic curve (blue) is not coincided well with the original contour (red) in sharp areas. Consequently, the second level with 25 control points is generated and the third level with 50 control points is generated as well. For some simple ROIs with smooth boundaries, few control points is enough to describe them. Hence, manual editing with such few control points may be enough to generate promising revision results. However, some anatomic structures in human body are apt to be complicated. For instance, some areas may be very sharp. Large control points need to be generated for manual editing for this case. As shown in Figures 5(c) and 5(d), the fitted Hermite cubic curve (blue) and original contour (red) are coincided well.

**Figure 6:**
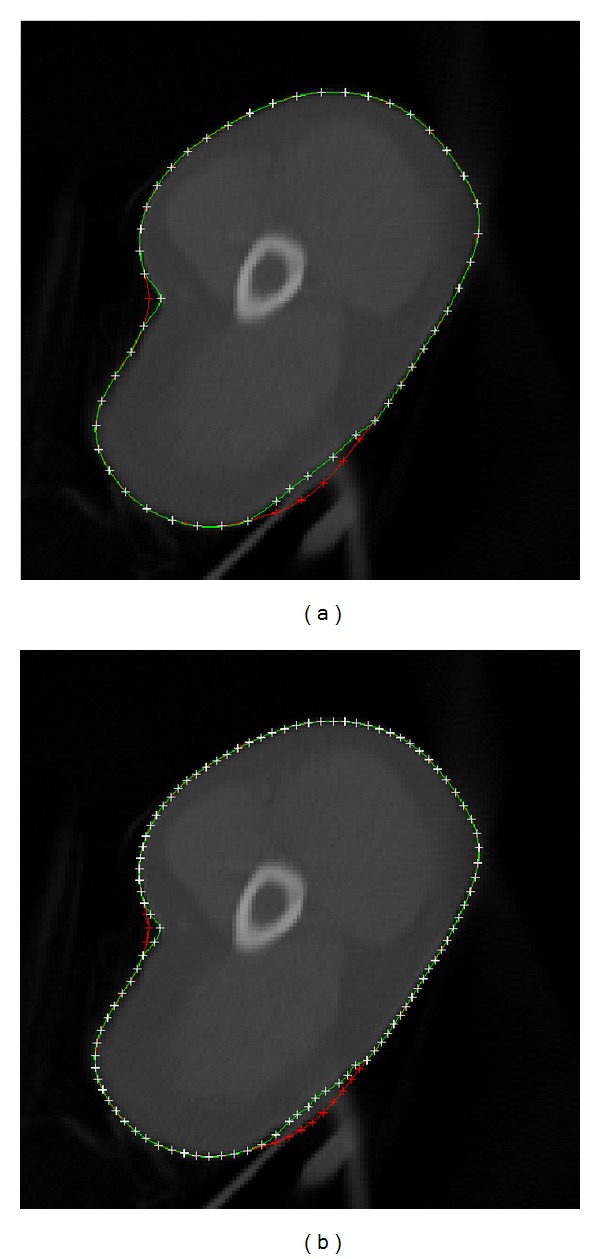
Manual editing: (a) manual editing with control points generated from the third level and (b) manual editing with control points generated from the fourth level. Red line shows the original contour generated by the snake model. Green line shows the final contour after manual editing. Red cross shows the related control points that are revised.

Figure 6 shows the results of manual editing to improve the contour from the snake model. It clearly shows that the final contour is very promising by manipulating several related control points. In Figure 6(a), only 6 related control points from the third level are revised to improve the contour generated by the snake model. Conversely, there are 12 related control points from the fourth level for the revision in Figure 6(b). Basically, more control points generated for manual editing will result in more accurate contour delineation, and the revised curve will accurately enclose the actual contour. However, large control points for revision will be cumbersome, time consuming, and boring for users. Therefore, the third level is suitable for manual editing, and the manual revision with several related control points can improve the contour of the snake model. If the clinician would like to generate more accurate boundary by manual editing, the fourth level would be better because more related control points can be revised to generate sharp contours.

The advantage of our method for number of control points is to find a compromise between efficiency and accuracy. Therefore, we design a strategy to generate control points hierarchically according to the length of region boundary. Firstly uniformly large spacing of control point mesh is generated when viable delineation errors of final contour of snake model are large. After manual revision with related control points, the overall delineation errors will be reduced. In order to refine the results further, the control point mesh is progressively refined. The control point spacing is halved at every step. With the revision of control point at different levels, the final contour will be generated to make the revised contour coincide with the actual contour. The process will be stopped until promising results are observed by clinicians. Obviously, it is flexible to represent complex shapes by progressive refinement. It makes it possible to revise small contours with more control points to guarantee the accuracy. Meanwhile, it is efficient and fast to revise large contours with relatively small control points.

## 3. Results and Discussion

The proposed interactive tool can be used to segment ROIs or delineate contours in images. To demonstrate the efficiency and robustness of our approach, we test the performance of the proposed method with kinds of medical images, such as CT images, MRI images, and ultrasound images in clinical applications. The delineation results in medical images using proposed method are presented, and the results of manual initialization, snake model, and manual editing are all shown, respectively. It is worthwhile to note that quantitative evaluation is generally difficult for real medial images since they contain complex anatomical structures and the manual segmentation by a human expert may be unavailable to be considered as the ground truth. Instead, qualitative results are mostly provided [2]. Therefore, a formal quantitative evaluation of the proposed method for clinical medical images is not contained in the paper due to the interactive nature of the method and the unknown ground truth data. Note that a basic implementation of the technique (such as Hermite curve, snake model, and multiscale curve editing) can be available from the first author by email (wu.zhouo@siat.ac.cn).

### 3.1. Clinical Image Test

**Figure 7:**
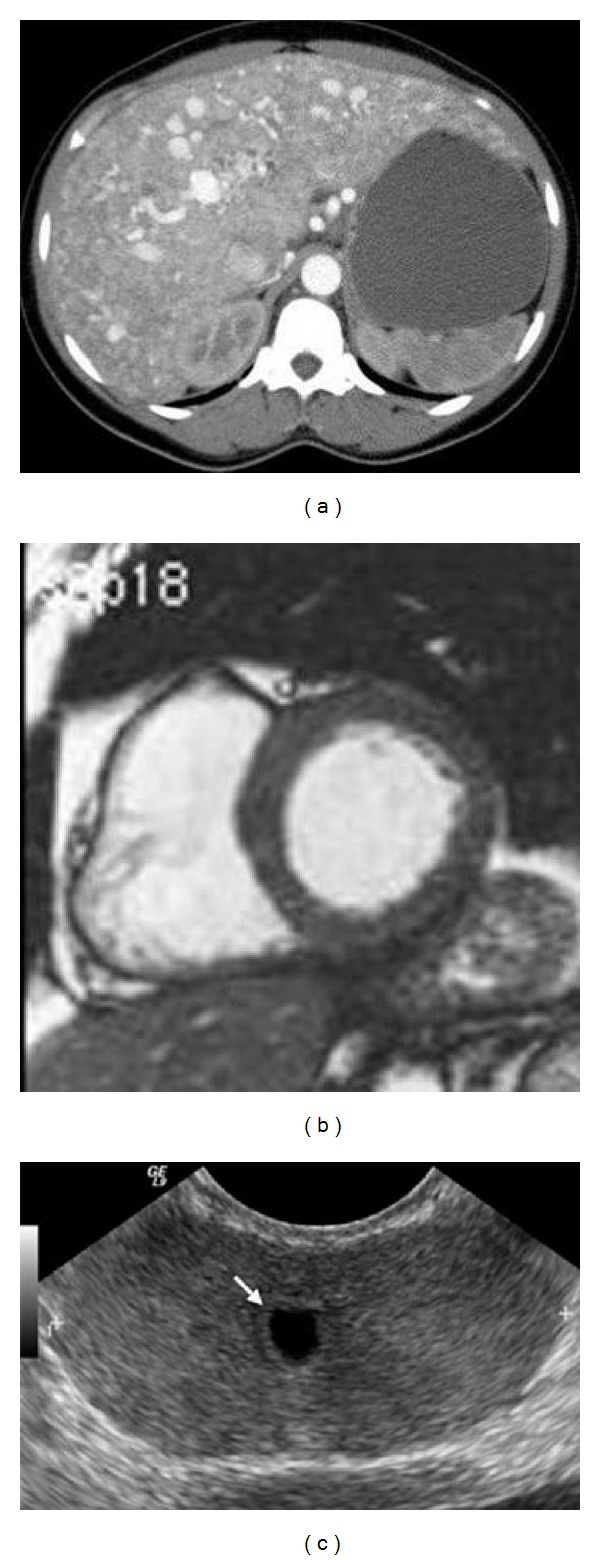
Clinical medical images in treatment planning: (a) contrast-enhanced axial CT scans through liver, (b) left ventricle in cardiac from MRI images and (c) transrectal ultrasound image of the prostate.

In clinical applications, most of the medical images are 3D, regions obtained in the slices are stacked to form a volumetric region after segmenting image slices individually. Without loss of generality, we take the case of 2D image registration for explanation. The CT image in Figure 7(a) is obtained from learning radiation website [21], which shows the contrast-enhanced axial CT scans through liver.  Figure 7(b) shows the left ventricle in cardiac from MRI images of the heart [22]. Development of contour detection techniques for the left ventricle is required to be able to reduce the total analysis time and to reduce the inter- and intraobserver variability associated with manual contour tracing.  Figure 7(c) shows the contour delineation result of a transverse image of the prostate in a young male that demonstrates a small midline cystic structure (arrow) representing a utricle cyst [23].

**Figure 8:**
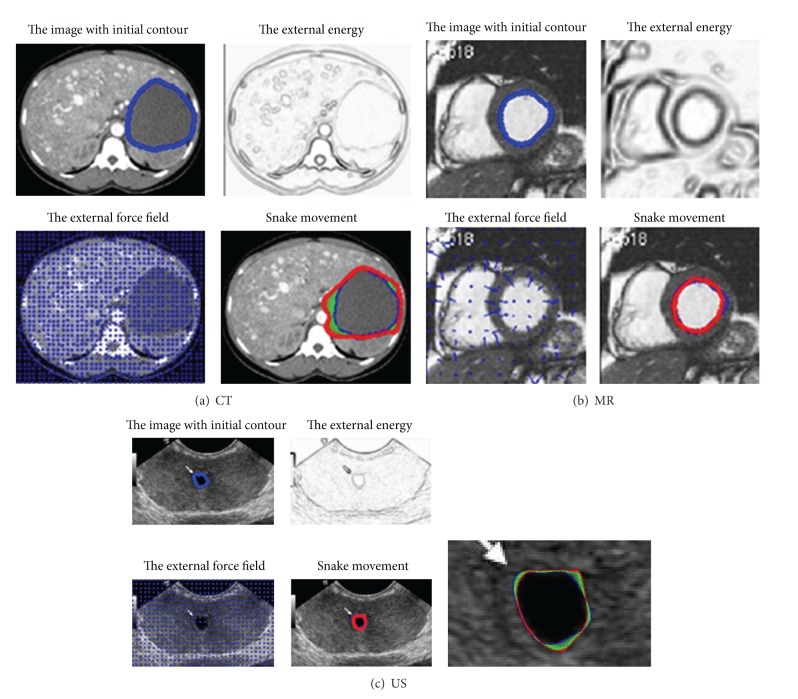
Contour delineation by basic snake model for CT, MR, and US images with optimal parameters. Blue shows the initial contour and red shows the final contour generated by the snake model. (a) CT, (b) MR, and (c) US. The parameter of maximum iteration number in the snake model is 300.

**Figure 9:**
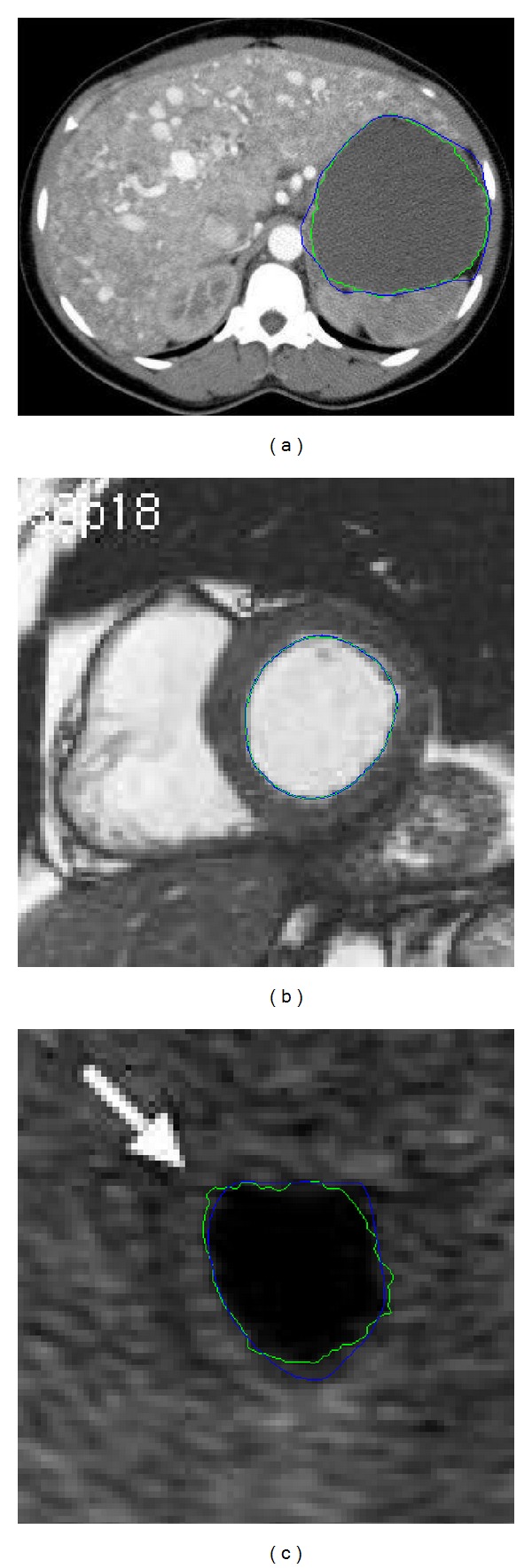
Contour delineation by the proposed method. Blue shows the contour generated by the snake model and green shows the final contour after multiscale curve editing (three levels). The final contour can be more flexible with multiscale control point revision. (a) CT, (b) MR, and (c) US. The parameter of maximum iteration number in the snake model of our framework is reduced to be 50.

Due to low resolution and low contrast of ultrasound images in addition to the speckle noise, either manual delineation methods or fully automatic delineation methods often contain errors for contour delineation. Although final contours obtained from the snake model are much smoother as shown in Figure 8, they are often away from the true boundaries. The reason is that sharp edges are smoothed out by the snake's internal energy which resists high curvature. For example in CT image, as shown in Figure 8(a), the boundary of the object is not salient and the result of snake model contains large errors. The results of the proposed method for medical images as shown in Figure 9 are visually good and promising. The refined contours are very close to the visual inspection or perceptual observations. Comparatively, the results obtained from the manual editing of the proposed method are robust and no obvious errors of final contours can be observed as shown in Figure 9. The manual editing of control points can reduce large boundary errors of the snake model, and obvious errors can be reduced by shifting several related control points. It is worthwhile to note that the segmentation of the blood pool/endocardium in Figure 9(b) after the snake model is perfect to some extent; it is optional for the clinicians to further apply Multiscale curve editing. The Multiscale curve editing has not been used for the MR image in Figure 9(b) due to the perfect delineation from the snake model.

The described interactive tool has been used to segment 2D medical images. Contour delineation of 2D images of size 512 × 512 pixels may take about few seconds. This time includes the initial manual drawing step, the snake model, and the interactive manual editing step on a laptop with CPU 3.3 GHz and 4 G RAM. The Hermite curve fitting in interactive manual revision step is real time with mouse action and takes no more than 0.02 seconds for each fitting due to the high efficiency of Hermite cubic curves. If the initial contour is far away from the actual contour, much consumed time is needed for the snake model. However, the contour initialization is close to the actual contour by the process manual initialization in our proposed method. Therefore, the snake model may take only few seconds to finish the contour evolution for the clinical images, as shown in Table 1. In general, the parameter of the maximum iteration number is often set at 200 or 300 for the snake model. Since the initial contour has been revised to be close to the desired contour, the maximum iteration number can be set much smaller than the original case in order to reduce iteration time. The parameter of maximum iterations for the snake model is 50 in the proposed framework. It is worthwhile to note that the time consuming of the snake model are obtained with initial contours which are close to actual boundaries. So the obtained time consuming is minimum because least time of convergence is required for contour evolution from the initial contour to the actual contour. In addition, the whole process for contour delineation is real time for users with mouse actions, no matter large ROIs or small ROIs in our experiments.

**Figure 10:**
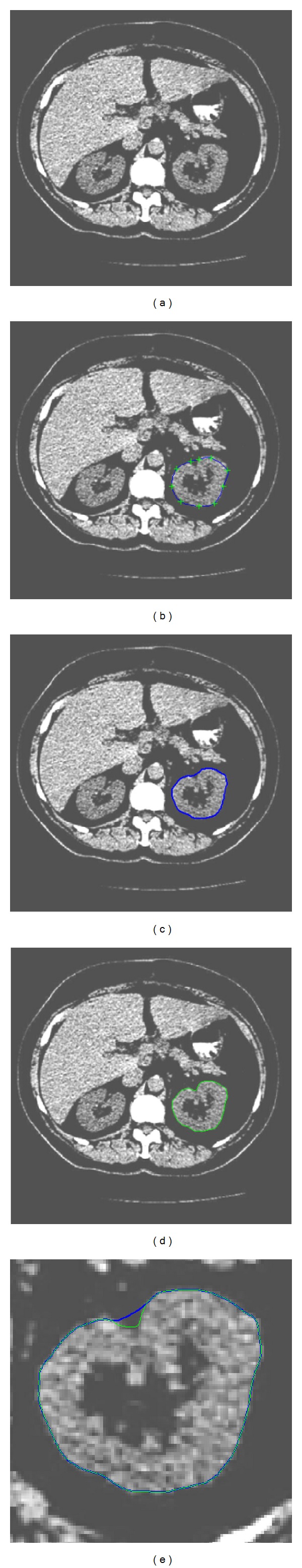
Delineation of the right kidney in an axial CT slice by the proposed method. (a) Original image, (b) initial contour and control points, (c) result of the snake model, (d) multiscale curve editing (two levels), (e) zoomed image. Blue shows the contour generated by the snake model and green shows the final contour after multiscale curve editing.

**Figure 11:**
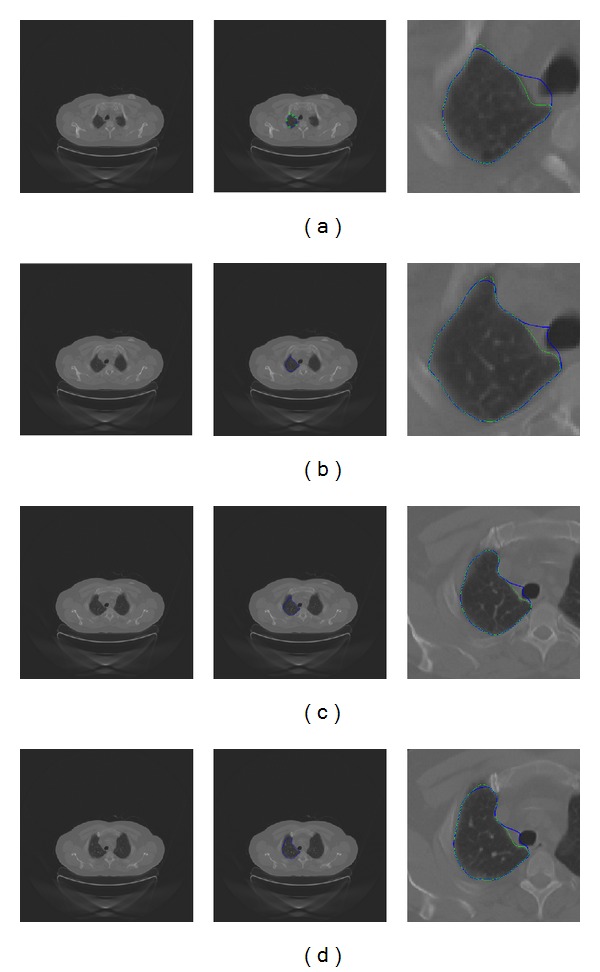
Tracking the boundary of the left lung in consecutive axial slices by the proposed method in 3D volumes. (a) Slice number 109, (b) slice number 108, (c) slice number 107, and (d) slice #106. The left column shows the original lung images in consecutive axial slices. The middle column shows the initial contour in corresponding axial slices. The right column shows the zoomed images of final contours obtained by the snake model (blue) and the multiscale curve editing (green, two levels). Note that the initial contour is only manually drawn in slice #109. The initial contour of other slices will be the final contour of its consecutive slice that has been delineated. For instance, the final contour of #109 will be the initial contour of slice number 108.

In order to demonstrate the effectiveness of the proposed method, we have further tested the clinical images by the proposed method.  Figure 10 shows the results of the proposed method for the segmentation of the right kidney in an axial CT slice. Sharp area of contours cannot be recovered well by the snake model; it is better for the clinicians to apply the multiscale curve editing to remove errors in sharp areas as shown in Figure 10(e). Note that the initial contour is revised manually to be close to the desired contour by dragging control points as shown in Figure 11(b).

Finally, we test our method in the segmentation of objects in a 3D volume. The proposed method was first used to segment an object of interest in an image. Then, the obtained contour was used to track the object boundary in subsequent image slices in a 3D volumetric image. Since the thickness of two slices is very small, the final contour in one slice can be treated as the initial contour in the next image slice automatically. Final contours obtained in the slices are stacked to form a volumetric object after segmenting image slices individually. If there are distinctive errors of segmentation in any slices, the proposed multiscale curve editing will be applied to refine the results of contours in slices individually. The top left image in Figure 11 shows the boundary of the left lung in consecutive axial slices by the proposed method in 3D volumes. The left column shows the original lung images in consecutive axial slices. The middle column shows the initial contour in corresponding axial slices. The right column shows the zoomed images of final contours obtained by the snake model (blue) and the Multiscale curve editing (green). It is evident to observe that the proposed method works well in consecutive axial slices in 3D volumetric images. Tracking the boundary of the left lung in consecutive axial slices will achieve segmentation of the left lung in 3D volumetric images. From the zoomed images in the right column of Figure 11, delineation results (blue) of the snake model for clinical images usually contain errors in sharp areas and low contrast regions. Therefore, multiscale curve editing is often required to improve the results of the snake model. The green contours obtained by the proposed method appear to be accurate and robust for the delineation.

### 3.2. Discussion

In general, snake model may not work well when the image is in low resolution and the true boundary is not distinctive, such as the above cases of clinical CT and ultrasound images as shown in Figure 7; the final contour of snake model will contain errors. Thus, the snake model is not reliable and robust in clinical applications. Moreover, the snake model is often time consuming when the start contour is not very close to the final solution and needs tenth of seconds or more for iteration optimization. Due to those reasons we develop such fast, efficient, and robust contour delineation approach for clinical applications.

Basically, the initial contour in our proposed method is manually drawn, and the process of manual initialization by shifting control points will make the initial contour close to the actual contour. Experimental results have shown that the time consuming of the snake model has been greatly reduced with the help of the process of manual initialization. Moreover, it is flexible to represent complex shapes by progressive refinement. Typically, more control points with fine spacing will be generated for the region of boundary with large curvature, and vice versa. The purpose of using equal distance and hierarchical multiscale manner is to make the process of manual revision simple and efficient in our proposed method. With the revision of control points at different levels, the final contour will be generated to make the revised contour coincide with the actual contour.

In this work, the manual revision process may be tedious for clinicians because of the involvement of much manual revision if the initial manual drawing is far away from the actual contour or required revision area is large from the active contour in clinical applications. Generally, the clinicians can control the manual drawing to make the initialization well, and the revision of few control points may be required. Compared with current automatic initialization methods [19], it is efficient but may be tedious for clinicians. In addition, the final segmentation results may not be much accurate in the multilevel manual revision process, since the visual observation for revision can only reduce or eliminate distinctive errors of segmentation viably. If the desired contour is unknown and complex, the multilevel manual revision can also be very tedious. To develop a method for automatic contour delineation in complex topology, noisy and low contrast images will be an essential task for our future work.

## 4. Conclusions

The purpose of this work has been to develop an interactive tool for kinds of medical image segmentation that can make the manual process highly efficient for clinical medical applications. Image segmentation has been achieved by using snake model and multiscale curve editing to obtain promising results. Our proposed technique allows users to freely and quickly improve contours by a simple mouse click and overcome the drawbacks of snake models for automatic contour delineation in clinical application. We believe that our proposed technique is applicable to various kinds of clinical applications for contour delineation or segmentation. In the future work, we will also consider 3D geometrical modeling of anatomical objects obtained by the boundary tracking with the proposed method for 3D volumetric images.

## Figures and Tables

**Table 1:** The consumed time of the Snake model with initial contours which are close to the actual boundaries (/seconds).

	Ct	Mr	Us
Snake model	2.69	1.17	1.92
